# Green & Sensitive pH-dependent Spectrofluorimetric Assay of Tamsulosin Hydrochloride and Tadalafil in their New Combined Formulation for Benign Prostatic Hyperplasia: Application to Spiked Human Plasma

**DOI:** 10.1007/s10895-022-02938-x

**Published:** 2022-05-16

**Authors:** Mona M. Abdel Moneim

**Affiliations:** Department of Pharmaceutical Chemistry, Faculty of Pharmacy, Pharos University in Alexandria, Alexandria, Egypt

**Keywords:** Green, pH-dependent spectrofluorimetry, Synchronous, Tamsulosin hydrochloride, Tadalafil and validation

## Abstract

Sensitive and green spectrofluorimetric methods were utilized for Tamsulosin Hydrochloride (TAM) and Tadalafil (TDL) assessment in bulk and their newly available combined mixture for benign prostatic hyperplasia and erectile dysfunction. The technique relies on measuring native fluorescence of TAM in 0.1 N HCl at 324 nm and TDL in 0.1 N NaOH at 348 nm due to their different fluorimetric behavior in acidic and basic media where TAM has no fluorescence in basic medium and vice versa. To achieve better regression, the spectra were derivatized allowing determination of TAM at 314 nm and TDL at 320 and 380 nm (peak to peak) by applying third and first derivative, respectively. In addition, pH-dependent “constant-wavelength synchronous” spectrofluorimetry was applied where TAM and TDL were determined at 218 nm in acidic medium and at 268 nm in basic medium, respectively. Finally, derivatizing the latter emission spectra allowed determination of TAM and TDL at 232 nm and at 262 and 278 nm (peak to peak), respectively. Acidic and basic emission spectra where scanned at λ_exc_ = 225 nm (for TAM assay) and at λ_exc_ = 247 nm (for TDL assay), respectively. Fluorescence–concentration plots were linear and the proposed methods were used for analysis of TAM and TDL combined laboratory prepared formulation. These procedures are green, sensitive and of low cost which make them suitable for quality control analysis of the two drugs. In addition, the high selectivity of the proposed methods was tested by successfully applying them for TAM and TDL assay in plasma samples.

## Introduction

The term “Lower urinary tract symptoms, LUTS” refers to all voiding and obstructive urinary symptoms which are known to be strongly related to benign prostatic hyperplasia (BPH). LUTS and BPH are known to increase with age so they are widespread in elderly men, above 50 years of age [1]. Recently, several researches also linked the high occurrence of erectile dysfunction (ED) for patients with BPH [2–4].

**Fig. 1:**
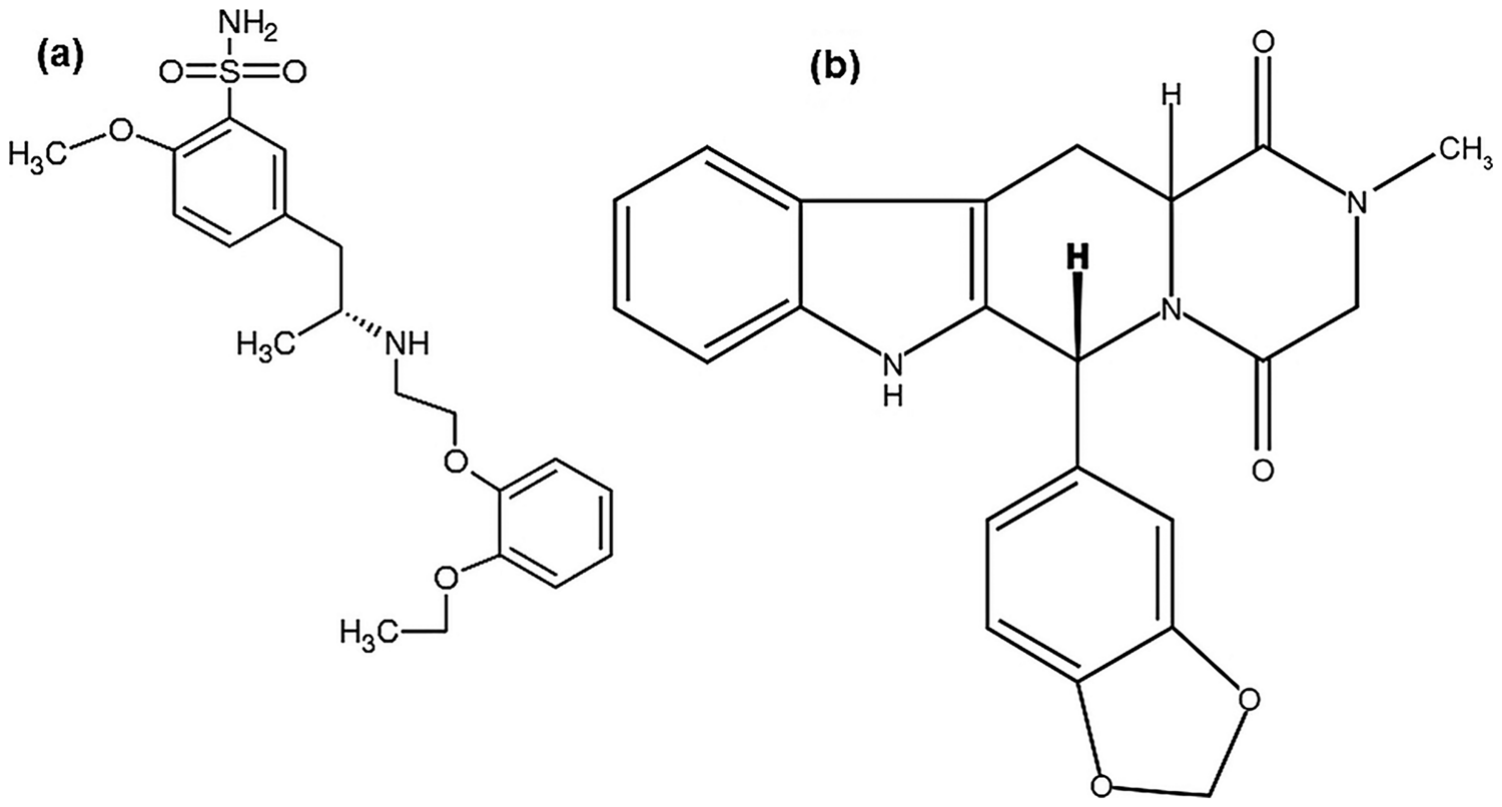
Chemical Structures of (**a**) Tamsulosin Hydrochloride (TAM) and (**b**) Tadalafil (TDL)

Tamsulosin HCl (TAM) is a selective α1A-adrenergic antagonist used as an effective treatment for BPH as it leads to relaxation of smooth muscle and urine obstruction relief [5]. TAM is chemically designed as 5-[(2R)-2-[[2-(2 Ethoxyphenoxy)ethyl]amino]-propyl]-2-methoxybenzenesulfonamide hydrochloride (Fig. 1a) [6, 7]. Tadalafil (TDL), a phosphodiesterase enzyme-5 inhibitor, is used originally to treat ED. But it has been recently approved to treat BPH patients with or without ED, where this can be attributed to its ability to reduce contraction of bladder neck and prostate and to improve urine flowage [8–10]. TDL is chemically known as (6*R*,12a*R*)-6-(1,3- Benzodioxol-5-yl)-2-methyl-2,3,6,7,12,12a-hexahydropyrazino[1’,2’:1,6]-pyrido[3,4-*b*] indole-1,4-dione (Fig. 1b) [6, 7].

Recently, TAM and TDL have been formulated as a combined formulation for treating BPH associated with ED. This binary combination, in many researches, proved to be safe and more effective than using each drug separately [4, 11]. In addition, it enhances patient compliance by administrating only one fixed dose combination with lower side effects [4, 10].

Several methods in the literature are reported for TAM or TDL assay as single components or with other drugs such as spectrophotometry [12, 13], spectrofluorimetry [14, 15], electrochemistry [16, 17], HPTLC [18, 19] and HPLC [20, 21].

TAM and TDL specifically together were analyzed in the recent literature by spectrophotometry [22], HPTLC [23, 24] and HPLC [24]. The aim of this study was to utilize spectrofluorimetry as it is green, low cost and highly sensitive technique with low solvent consumption for TAM and TDL assay as a newly formulated binary mixture.

This study shows the multiplicity of fluorimetry in utilizing different spectrofluorimetric behavior of drugs in different pHs. Also, it put emphasizes on the sensitivity that can be achieved without breaching the rules of green analysis. The proposed spectrofluorimetric methods are dependent on the difference in relative fluorescence intensity (RFI) of TAM and TDL in acidic and basic media. Direct and derivative pH-dependent spectrofluorimetric and synchronous spectrofluorimetric measurements were applied and compared in terms of their sensitivity and selectivity. In addition, both validation and analysis of this binary combination in laboratory prepared tablets and spiked human plasma samples were fulfilled.

## Experimental

### Spectrofluorimetric Apparatus

Cary Eclipse Spectroflourimeter (G9800A, Agilent technologies) and quartz cells of 1 cm were used for all spectrofluorimetric measurements. It is also connected to a computer with Cary Eclipse software.

### Reagents and Chemicals

- TAM (99.99%) and TDL (100.06%) were brought from Sigma Aldrich, USA.
- Methanol was HPLC grade (Sigma-Aldrich Chemie GmbH, Buchs, Switzerland).
- NaOH and HCl used in the study were Analar grade (El-Nasr Pharmaceutical Chemicals, Cairo, Egypt).
- Human plasma sample was obtained from Alexandria blood bank (Alexandria, Egypt).

TAM and TDL newly marketed dosage form is unavailable in our commercial markets. Thus, laboratory-prepared tablets containing 0.4 and 5 mg of TAM and TDL, respectively, per tablet were used as their concentrations in the marketed tablets. The tablets were prepared using commonly used tablet fillers kindly supplied by Pharco Pharmaceuticals Co., Alexandria, Egypt, composed of maize starch, microcrystalline cellulose (avicel), magnesium sterate, hydroxyl propyl methyl cellulose and colloidal silica (aerosil).

### Standard Solutions and Calibration Standards

Standard stock solutions of TAM and TDL were prepared in methanol, separately, with concentrations of 1000 μg/mL. These stock solutions were stable for a minimum of two weeks at 4 °C away from light. Aliquots (μLs, using micropipette) from TAM and TDL standard stock solutions (1000 μg/mL, each), corresponding to Table 1 linearity range, were transferred into two separate sets of 10-mL volumetric flasks and adjusted all to contain an overall of 200 μL methanolic content. TAM set was completed with 0.1 N HCl to the mark (for TAM determination) and the other TDL set was completed to mark with 0.1 N NaOH (for TDL determination).

**Table 1:** Regression parameters of the proposed spectrofluorimetric methods for determination of TAM with TDL

**Method**	**Direct fluorimetric method**		**Derivative fluorimetric method**		**Direct Synchronous method**		**Derivative Synchronous method**	
**Parameter**	**TAM**	**TDL**	**TAM**	**TDL**	**TAM**	**TDL**	**TAM**	**TDL**
Linearity µg/mL	1–35	1–32	1–35	1–32	0.5–10	0.5–15	0.5–10	0.5–15
Intercept, a	29.67	67.12	6.15	10.66	0.70	-8.088	-13.80	1.08
Slope, b	24.29	10.49	5.59	3.48	13.93	13.87	-13.83	2.64
LOQ, µg/mL	6.39	11.14	5.18	3.76	3.25	1.25	1.07	1.68
LOD, µg/mL	2.11	3.67	1.71	1.24	1.07	0.41	0.35	0.56
Correlation coefficient (r)	0.9981	0.9941	0.9992	0.9991	0.9913	0.9995	0.9990	0.9991
S_a_	15.52	11.70	2.90	1.31	4.52	1.74	1.48	0.44
S_b_	0.86	0.66	0.13	0.07	13.93	0.25	0.30	0.06
S_y/x_	25.80	16.59	4.04	1.86	7.15	2.97	2.35	0.68
F	801.34	249.97	1886.34	2249.26	228.77	3120.68	2101.10	2172.22
Significance F	9.68 × 10^–5^	5.50 × 10^–4^	2.69 × 10^–5^	2.06 × 10^–5^	1.11 × 10^–4^	1.26 × 10^–5^	1.35 × 10^–6^	1.27 × 10^–6^

*LOQ* limit of quantitation, *LOD* limit of detection, *S*_*a*_ standard deviation of intercept, *S*_*b*_ standard deviation of slope, *S*_*y/x*_ standard deviation of residuals

### Synthetic Mixtures

Aliquots of TAM and TDL were transferred from their corresponding standard stock solutions into a set of 10-mL volumetric flasks to achieve linearity ranges stated in Table 1. All volumetrics were adjusted to have a constant volume of methanol of 200 μL, by adding the appropriate volume of methanol on the drugs’ aliquots in each flask. The volumetric flasks were then completed with 0.1 N HCl to the mark (for TAM determination) one time and another time with 0.1 N NaOH (for TDL determination).

### Applied Procedures

#### Construction of Calibration Curves

For each calibration standard of TAM and TDL prepared in “Standard Solutions and Calibration Standards” section, the emission spectra (1 nm interval) were recorded with excitation at 225 and 247 nm for acidic and basic solutions, respectively. All emission spectra were corrected with methanolic HCl or NaOH blank signal and processed using Microsoft Excel®.

##### Direct Fluorimetric Method

The amplitude values of the acidic emission spectra in 0.1 N HCl at 324 nm, were used for TAM determination (where TDL shows no RFI). Also, amplitude values of the alkaline emission spectra in 0.1 N NaOH at 348 nm were measured for the determination of TDL (where TAM shows negligible RFI).

##### Derivative Fluorimetric Method

The third derivative values of the acidic emission spectra were computed at 314 nm for TAM determination (TDL zero crossing) using Δλ of 3 nm. Also, the first derivative values of the alkaline emission spectra were computed by measuring peak to peak at 320 and 380 nm (TAM zero crossing) for the determination of TDL using Δλ of 3 nm.

##### Direct Synchronous Method

Synchronous spectra of both drugs in acidic and basic solvents were scanned in range of 190–400 nm keeping 90 nm constant difference between the two monochromators of excitation and emission. Values of the synchronous emission spectra in HCl at 218 nm, were used for TAM determination (where TDL shows negligible RFI). Also, values of the synchronous emission spectra in NaOH at 268 nm, were computed for determination of TDL.

##### Derivative Synchronous Method

The first derivative values of the acidic synchronous spectra were measured at 232 for the determination of TAM (TDL zero crossing) and in 0.1 N NaOH, peak to peak measurements were done at 262 and 278 nm for the determination of TDL, all using Δλ of 3 nm.

#### Analysis of Synthetic Mixtures

TAM and TDL mixtures with different ratios were measured against suitable blank and their emission spectra were computed by the four methods to assay the concentration of each drug and its recovery in each mixture.

#### Analysis of Laboratory Prepared Tablets

Ten laboratory-prepared tablets were weighed and powdered well and a weight equivalent to 2 and 25 mg TAM and TDL, respectively was transferred by methanol, into a 25 mL volumetric flask. After sonication for 15 min, dilution was made with methanol to volume and filtered using Whatman No-1 filter paper. A 125 μL portion of the prepared sample solution and 75 μL methanol were added into two 10-mL volumetric flasks, then one of them was diluted to volume with 0.1 N HCl and the other with 0.1 N NaOH. The concentrations achieved were 1 and 12.5 μg/mL of TAM and TDL, respectively. The procedures were completed on the prepared solutions as previously described.

#### Analysis of Spiked Human Plasma Samples

Aliquots from TAM and TDL methanolic stock solutions were added to 1 mL plasma samples to prepare five mixtures with different ratios (1: 12.5, 1: 6, 1: 1, 10: 1, and 1: 5 for TAM: TDL). The solutions were vortexed for 3 min after being completed to a total volume of methanol = 5 mL and the tubes were then centrifuged at 4000 rpm (15 min). A 200 μL of the supernatant was carefully transferred into 10 mL volumetric flasks and completed to volume with either HCl or NaOH where the analysis was continued as described previously against blanks.

## Result and Discussion

TAM and TDL are recently co-formulated together in a newly marketed dosage form where TAM is the minor component with concentration of 0.4 mg in comparison to TDL (5 mg). In pharmaceutical industry and quality control analysis, the development of new assay techniques to analyze newly challenging co-formulated and marketed drug products should always be addressed with its challenges, due to its importance.

Usually analysts resolve multi component mixtures using chromatographic methods as HPLC [25] and HPTLC [26] using organic toxic solvents with large amounts. However, spectrofluorimetry is a sensitive, green and low cost method that is highly favored in pharmaceutical analysis of complex mixtures. For strong spectral overlaps, derivative spectrofluorimetry is usually applied allowing simultaneous determination of complex mixtures as it improves minor spectral characteristics [12, 14, 27, 28].

Meanwhile, in constant-wavelength synchronous fluorimetry: the emission spectra are measured by synchronously scanning excitation and emission monochromators with a constant ∆λ offering much higher spectral resolution, selectivity and sensitivity because it gives sharp and narrow spectra.

**Fig. 2:**
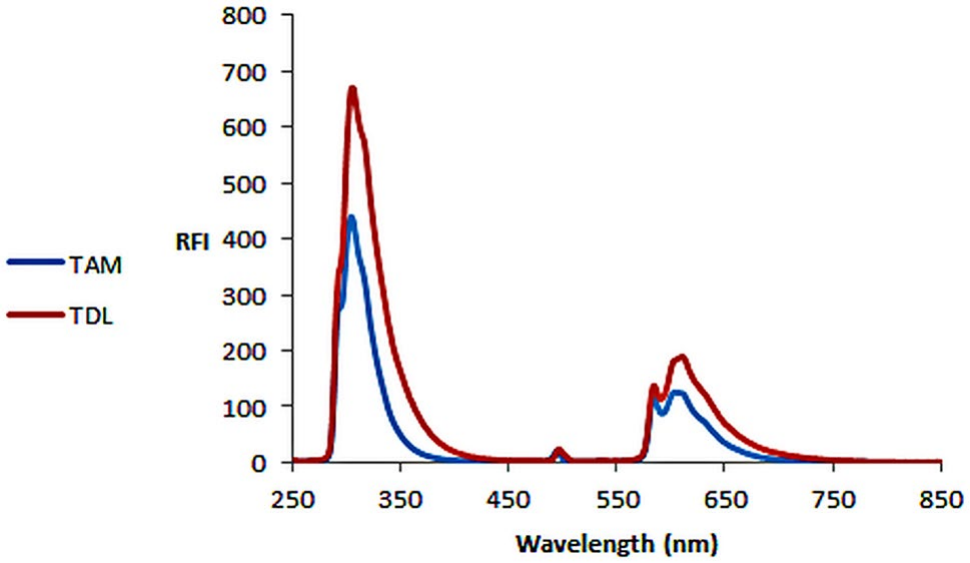
Emission spectra of TAM and TDL upon excitation at 250 nm in methanol

Upon preliminary scanning of TAM and TDL emission spectra (Fig. 2), it was noticed the severe similarity in the fluorimetric spectra and behavior of TAM and TDL in several solvents as methanol, water and acetonitrile which hinders their simultaneous determination even after mathematical transformations as derivative or synchronous or Fourier convolution. In addition, TAM being the minor component in the pharmaceutical dosage form ratio, its spectrofluorimetric spectrum was completely covered by that of TDL which is very typical to it.

**Fig. 3:**
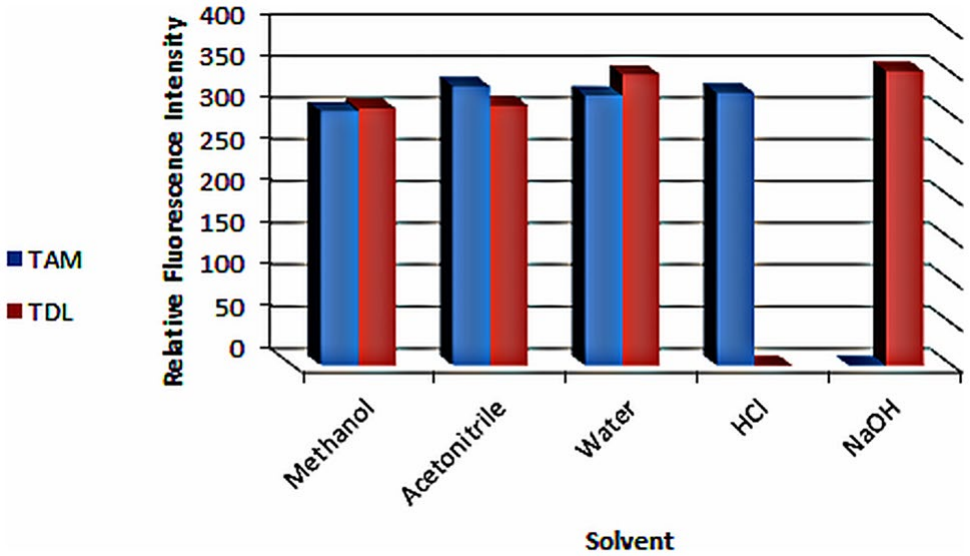
Effect of different diluting solvents on RFI of 12 and 31.25 µg/mL TAM and TDL, respectively (each at its best λ_exc_)

By noticing the spectral behavior of the two concerned drugs in several solvents (Fig. 3), it was noticed that TAM had shown acceptable RFI in acidic medium using 0.1 N HCl and no RFI in alkaline medium using 0.1 N NaOH and TDL had opposite behavior. Thus, the proposed spectrofluorimetric methods took use of the different spectrofluorimetric behavior of both drugs in different pH and resolved there strong overlapping without preliminary separation, or tedious mathematical calculations. This was achieved by simply measuring TAM in acidic medium and TDL in basic medium in presence of each other without interference and with satisfactory selectivity, accuracy and precision, even in plasma samples.

### Method Development and Optimization

#### Direct Fluorimetric Method

**Fig. 4:**
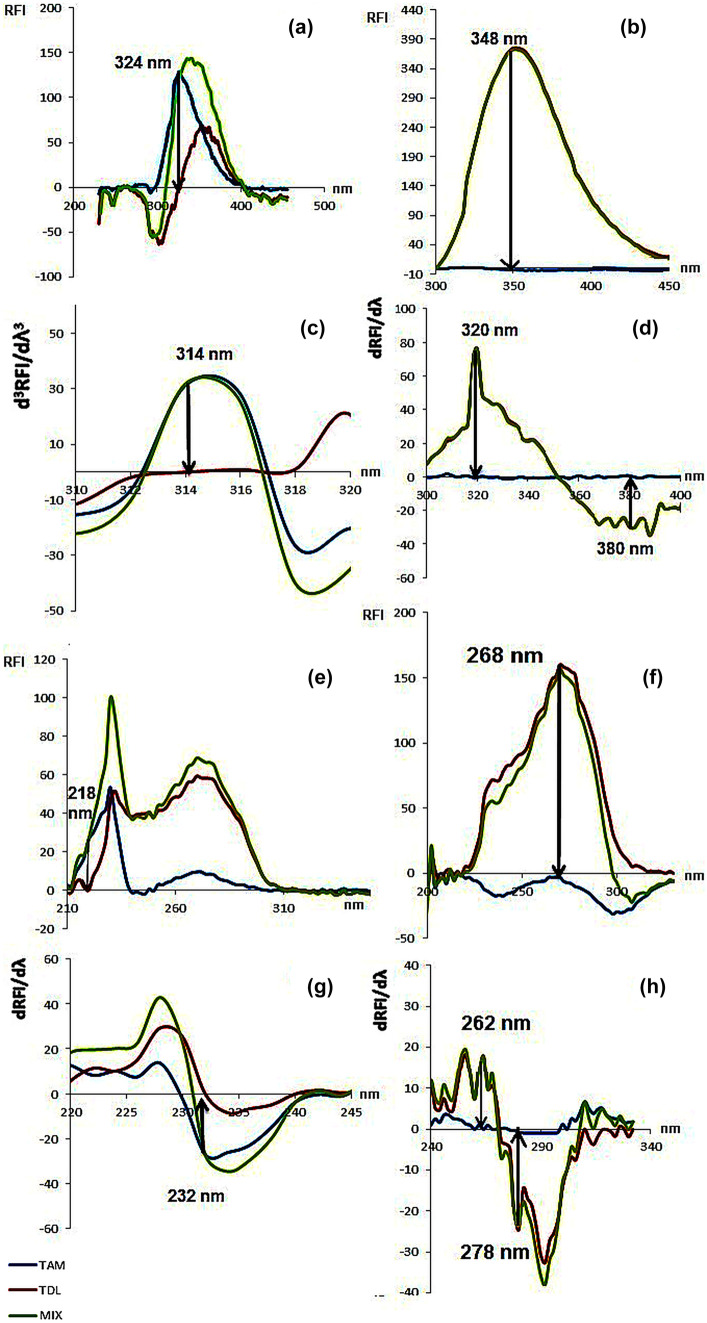
Emission spectra of 2.5 μg/mL TAM, 31.25 μg/mL TDL and their mixture in (**a**) 0.1 N HCl (λ_exc_ = 225 nm), (**b**) 0.1 N NaOH (λ_exc_ = 247 nm), (**c**) 0.1 N HCl (D^3^ using Δλ = 3 nm) and (**d**) 0.1 N NaOH (D^1^ using Δλ = 3 nm) and constant wavelength synchronous spectra (Δλ = 90 nm) of 1 μg/mL TAM, 12.5 μg/mL TDL and their mixture in (**e**) 0.1 N HCl, (**f**) 0.1 N NaOH, (**g**) 0.1 N HCl (D^1^ using Δλ = 3 nm) and (**h**) 0.1 N NaOH (D^1^ using Δλ = 3 nm). (TAM  , TDL  and MIX  )

TAM and TDL presents very similar native fluorescence. Several solvents were tried but the only difference between the emission spectra of both drugs was in acidic and basic media upon excitation at their corresponding λ_ex_ (225 nm for TAM acidic spectra and 247 nm for TDL basic spectra) as explained earlier and shown in Fig. 3. The acidic emission spectrum of TAM was measured at 324 nm where TDL (major component) had zero-crossing. Meanwhile, the basic emission spectrum of TDL was measured at 348 nm for TDL determination (TAM has no RFI in basic medium) (Fig. 4a, b).

#### Derivative Fluorimetric Method

Derivative fluorimetry is known to improve resolution of overlapping bands and increase compounds’ spectral characteristics [12, 14, 27, 28]. In case of TAM and TDL binary mixture, the resulted acidic spectra after third derivative revealed that TAM could be determined by peak measurement at 314 nm without any interference from TDL, as the latter showed zero crossing. On the other hand, TDL could be analyzed by peak to peak measurements at 320 and 380 nm with zero crossing of TAM at these two wavelengths using first derivative of the alkaline spectra (Fig. 4c, d).

For choosing best wavelength interval (Δλ) for TAM and TDL assay, different Δλs were tried from 3–11 nm to derivatize the data. The Δλ giving best linearity, sensitivity and percentage recoveries was Δλ of 3 nm.

#### Direct Synchronous Method

Synchronous “constant-wavelength” mode was also tried to improve spectral resolution and allow measurement of the two drugs directly without mathematical derivatization where the two monochromators are simultaneously scanned with constant Δλ. Several Δλs were studied from 10 to 100 nm. For obtaining the synchronous spectra of these two drugs with maximum spectral resolution and sensitivity for TAM (the minor component), Δλ = 90 nm was found suitable and gave reasonable response for both drugs at their dosage form ratio.

Synchronous emission spectra had advantages over conventional spectrofluorimetry in giving much simpler narrow spectra, allowing quantitative determinations with higher sensitivity than the first two methods (Table 1). However, applying synchronous measurements on methanolic solutions of both drugs revealed highly overlaid spectra. Thus, synchronous technique was applied also on solutions of both drugs in basic and acidic media to create a difference in their fluorimetric behavior. The emission values at 218 nm in acidic medium and at 268 nm in basic medium were used for the determination of TAM and TDL, respectively, in their mixture (Fig. 4e, f).

#### Derivative Synchronous Method

The first derivative values of the synchronous spectra in HCl were measured at 232 nm for the determination of TAM and in NaOH, peak to peak measurements were done at 262 and 278 nm for the determination of TDL, all using Δλ of 3 nm. (Fig. 4g, h).

The selected wavelengths for each drug showed zero contribution from the other component in the mixture allowing its accurate determination. The optimization of Δλ was also studied in the same range (3–11 nm) and Δλ = 3 nm was also chosen.

### Effect of pH

**Fig. 5:**
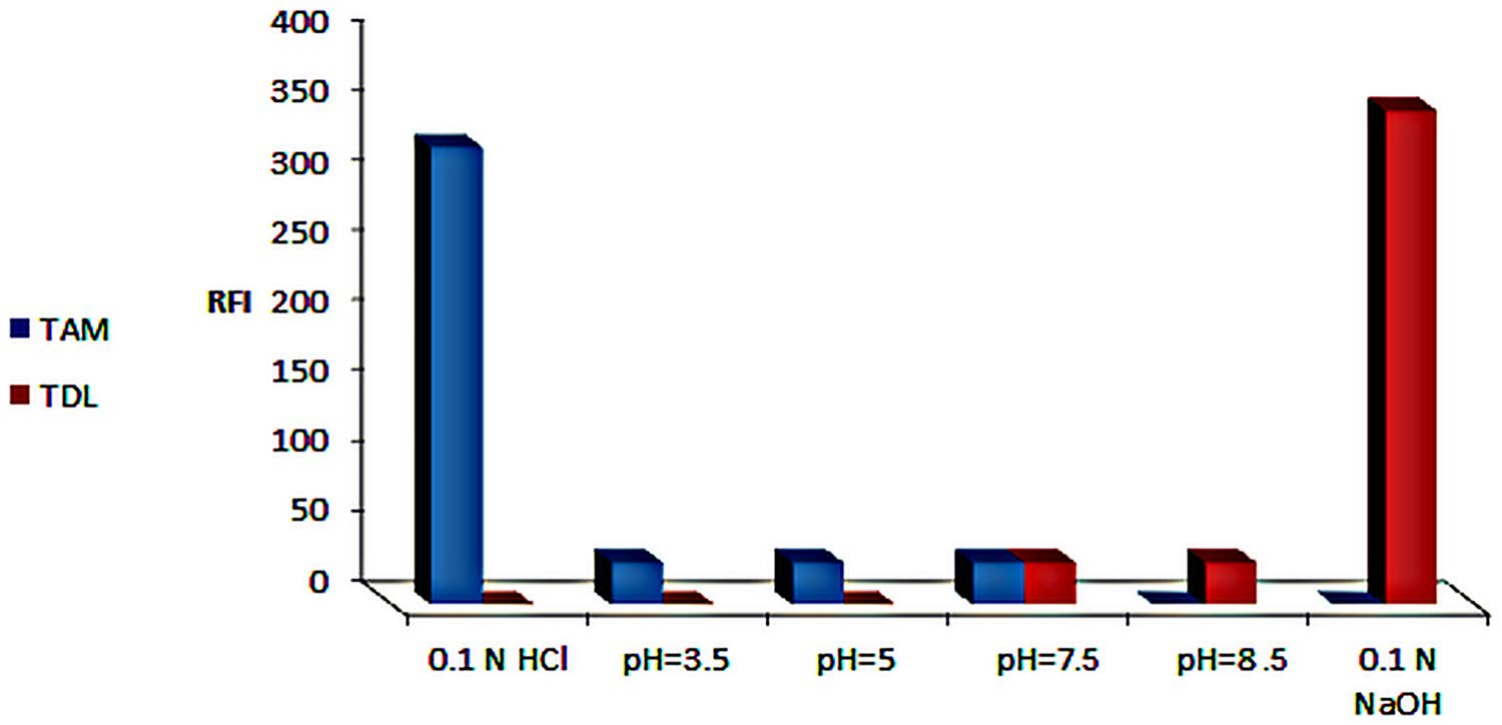
Effect of pH on RFI of 12 and 31.25 µg/mL TAM and TDL, respectively

The pH impact on TAM and TDL fluorescence was studied using various types of buffers such as 0.2 M acetate buffer (pH 3.5–6) and 0.2 M borate buffer (pH 6.5–8.5), in addition to 0.1 N HCl and 0.1 N NaOH. The buffer usage had negative impact on the RFI over the entire studied pH range. It was found that maximum RFI was obtained only in 0.1 N HCl for TAM and 0.1 N NaOH for TDL (Fig. 5).

### Validation

The methods were validated according to ICH guidelines [29]:

#### Linearity and Sensitivity

Linearity was checked for all the proposed methods within the ranges stated in Table 1 including their dosage form ratio (1:12.5, TAM: TDL)**.** Statistical assessment of the results shows acceptable correlation coefficient (r) values, small values of standard deviation of residuals (S_y/x_), standard deviation of intercept (S_a_) and standard deviation of slope (S_b_), in addition to high F-values with low significance F-values (Table 1). LOD and LOQ were calculated according to Miller: LOD = 3.3S_a_/b and LOQ = 10S_a_/b [30]. Derivatizing the emission spectra improved the regression and correlation coefficients of the data (increase in r values). Also, the synchronous methods showed better sensitivity and lower LOD and LOQ. The proposed methods are spectrofluorimetric methods for analysis of TAM and TDL with much better sensitivity compared with spectrophotometric methods. Also, they are superior to chromatographic methods in having simpler steps, less time, lower cost and less solvent consumption making them green and eco-friendly.

#### Accuracy, Precision and Selectivity

Acceptable accuracy and precision were indicated by the % recoveries (± 2%) and low RSD% values (≤ 2%) after analysis of synthetic mixtures of TAM and TDL at different ratios as stated in Table 2. The samples were processed on one day and on three different days (n = 3). Successful selective determination of TAM and TDL with no interference from each other, in different ratios, also shows the selectivity of the proposed methods.

**Table 2:** Evaluation of intra‐ and inter‐day precision and accuracy of the proposed spectrofluorimetric methods for determination of TAM with TDL

**Method**		**Direct fluorimetric method**		**Derivative fluorimetric method**		**Direct Synchronous method**		**Derivative Synchronous method**	
**(a) Accuracy and Intra-day precision (repeatability)**									
**Concentration** **(µg/mL)**		Mean % Recovery ± %RSD ^*^							
**TAM**	**TDL**	**TAM**	**TDL**	**TAM**	**TDL**	**TAM**	**TDL**	**TAM**	**TDL**
1	12.5	100.66 ± 1.30	100.30 ± 1.04	101.01 ± 1.44	100.45 ± 0.75	99.36 ± 0.65	100.11 ± 0.67	102.09 ± 0.59	99.60 ± 0.81
2.5	15	100.89 ± 0.87	100.89 ± 0.89	100.45 ± 0.99	102.01 ± 1.09	99.55 ± 0.89	101.07 ± 0.78	98.29 ± 1.00	99.45 ± 1.33
10	10	99.36 ± 1.12	99.32 ± 1.33	100.91 ± 1.05	101.74 ± 1.55	100.44 ± 1.09	101.66 ± 1.04	99.15 ± 1.02	99.30 ± 1.00
10	1	101.35 ± 0.89	101.55 ± 0.78	101.43 ± 1.20	101.49 ± 0.88	99.64 ± 1.14	98.23 ± 1.55	100.37 ± 1.33	99.80 ± 1.07
1	5	100.40 ± 1.00	98.71 ± 1.09	100.25 ± 1.66	99.56 ± 1.35	99.09 ± 1.20	99.51 ± 0.91	100.67 ± 0.80	100.26 ± 0.88
**Grand mean**		**100.53 ± 1.04**	**100.15 ± 1.03**	**100.81 ± 1.27**	**101.05 ± 1.12**	**99.62 ± 0.99**	**100.12 ± 0.99**	**100.11 ± 0.95**	**99.68 ± 1.02**
**(b) Accuracy and Inter-day precision**									
**Concentration (µg/mL)**		Mean % Recovery ± %RSD ^*^							
**TAM**	**TDL**	**TAM**	**TDL**	**TAM**	**TDL**	**TAM**	**TDL**	**TAM**	**TDL**
1	12.5	100.45 ± 0.60	100.11 ± 0.90	98.21 ± 0.87	101.50 ± 0.90	100.75 ± 0.66	99.45 ± 1.80	98.65 ± 0.98	100.20 ± 1.22
2.5	15	100.39 ± 1.03	100.54 ± 0.77	99.45 ± 1.07	99.82 ± 0.70	99.38 ± 0.91	99.99 ± 1.90	99.21 ± 0.84	101.97 ± 0.86
10	10	100.65 ± 0.78	101.27 ± 1.01	101.55 ± 1.55	100.70 ± 1.01	101.25 ± 1.09	98.67 ± 1.06	99.39 ± 0.91	99.35 ± 1.09
10	1	101.20 ± 0.96	99.67 ± 1.50	100.33 ± 0.80	100.43 ± 1.33	99.71 ± 1.10	101.20 ± 0.90	100.09 ± 1.05	100.65 ± 1.41
1	5	101.35 ± 1.02	98.99 ± 1.35	99.44 ± 1.05	101.25 ± 0.99	101.55 ± 1.90	101.77 ± 0.66	101.60 ± 1.01	99.99 ± 0.77
**Grand mean**		**100.81 ± 0.88**	**100.12 ± 1.12**	**99.80 ± 1.07**	**100.74 ± 0.99**	**100.06 ± 1.13**	**100.22 ± 1.26**	**99.79 ± 0.96**	**100.43 ± 1.07**

^*^Mean ± percentage relative standard deviation of three determinations

#### Solutions Stability

The prepared solutions of TAM and TDL were found to be stable when left at room temperature for at least 6 h which was sufficient for all measurements (RSD% ≤ 2%).

### Assay of Laboratory Prepared Tablets

The proposed methods were applied for TAM and TDL analysis in laboratory prepared tablets without interference. The obtained recoveries of the studied drugs were in agreement with the label claims (Table 3). A comparison between the proposed method and another reported spectroscopic method was done by Student’s t- and variance ratio F- tests. Calculated values did not exceed theoretical ones (at 95% confidence level) which prove there is no significant difference between the developed fluorimetric methods and the comparison method (Table 3).

**Table 3:** Assay results for determination of TAM & TDL in their laboratory-prepared pharmaceutical preparation containing 0.4 mg TAM & 5 mg TDL by the proposed spectrofluorimetric methods

**Test**	**% Found ± SD** (n = 5)				
	**TAM**				
	**Direct fluorimetric method**	**Derivative fluorimetric method**	**Direct Synchronous method**	**Derivative Synchronous method**	**Comparative Spectrophotometric method** [22]
	101.61 ± 0.80	101.27 ± 1.24	100.86 ± 1.46	101.44 ± 0.99	101.28 ± 0.96
Students’ t- test (t)^*^	0.59	0.99	0.64	0.82	……………….
Variance ratio F- test (F)^*^	0.91	0.75	0.32	0.78	……………….
	**TDL**				
**Test**	**Direct fluorimetric method**	**Derivative fluorimetric method**	**Direct Synchronous method**	**Derivative Synchronous method**	**Comparative Spectrophotometric method** [22]
	100.19 ± 1.01	101.12 ± 1.61	100.29 ± 0.85	100.45 ± 1.08	99.71 ± 1.14
Students’ t- test (t)^*^	0.52	0.68	0.41	0.35	……………….
Variance ratio F- test (F)^*^	0.99	0.40	0.74	0.92	……………….

^*^Theoretical values of *t* and *F* are 2.31 and 6.39, respectively, at 95% confidence limit

### Analysis of TAM and TDL in Spiked Human Plasma Samples

TAM and TDL assay in spiked plasma samples was performed in different ratios to test methods’ selectivity with just simple protein precipitation step. As shown in Table 4, high % recoveries (95–105%) and low % RSD values (≤ 2%) proved the ability and high selectivity of these fluorimetric methods to assay TAM and TDL in spiked human plasma with minimal solvent consumption and cost. (Table 4).

**Table 4:** Application of the proposed methods for the determination of TAM and TDL in spiked human plasma

**Method**		**Direct fluorimetric method**		**Derivative fluorimetric method**		**Direct Synchronous method**		**Derivative Synchronous method**	
**Concentration after extraction** **(µg/mL)**		Mean % Recovery ± %RSD ^*^							
**TAM**	**TDL**	**TAM**	**TDL**	**TAM**	**TDL**	**TAM**	**TDL**	**TAM**	**TDL**
1	12.5	96.67 ± 1.91	98.40 ± 1.07	97.36 ± 1.50	102.89 ± 1.85	97.74 ± 0.97	99.90 ± 1.65	96.11 ± 1.50	101.43 ± 1.12
2.5	15	96.01 ± 1.11	97.23 ± 0.99	98.78 ± 1.77	102.04 ± 1.50	99.65 ± 1.82	101.78 ± 1.55	98.49 ± 1.55	100.98 ± 1.30
10	10	100.55 ± 1.45	100.19 ± 1.22	99.44 ± 1.78	100.99 ± 0.80	101.35 ± 1.42	103.40 ± 1.09	100.01 ± 0.90	99.67 ± 0.96
10	1	102.98 ± 1.59	101.95 ± 1.35	100.90 ± 1.99	96.40 ± 0.95	100.25 ± 1.40	98.64 ± 1.71	100.99 ± 1.11	96.40 ± 1.96
1	5	103.20 ± 1.92	97.15 ± 0.80	95.55 ± 1.20	96.53 ± 1.66	96.33 ± 1.32	96.46 ± 1.13	105.98 ± 1.30	98.11 ± 1.32

^*^Mean ± percentage relative standard deviation of three determinations

## Conclusion

New analytical methods must be available for analysis of newly marketed pharmaceutical combinations for treating different conditions. This is the case for TAM and TDL recently developed co-formulation for treating benign prostate hyperplasia and erectile dysfunction in elderly men. Four simple, selective and time saving fluorimetric methods were established for resolving this mixture in its challenging ratio. Green analytical chemistry is based on minimal use of expensive and toxic solvents and instruments consuming large amount of energy (like HPLC). The proposed fluorimetric methods fulfilled the criteria of green chemistry as it only used simple solvents as HCl and NaOH with the spectrofluorimeter. Derivatizing the spectra in general improved the data’s regression and synchronizing the spectra with constant wavelength difference changed the characteristics of the emission spectra which allowed more sensitive determination of the two drugs. The proposed methods could analyse the two drugs in their pure drugs substances, their combined laboratory made dosage form and spiked human plasma samples.

## Data Availability

The datasets used and/or analysed during the current study are all available in the manuscript in details, for more information contact the corresponding author.
